# Aldehyde-induced DNA-protein crosslinks- DNA damage, repair and mutagenesis

**DOI:** 10.3389/fonc.2024.1478373

**Published:** 2024-09-12

**Authors:** Thomas Blouin, Natalie Saini

**Affiliations:** Department of Biochemistry and Molecular Biology, Medical University of South Carolina, Charleston, SC, United States

**Keywords:** DNA protein crosslink, aldehydes, DNA damage, DNA repair, genome instability

## Abstract

Aldehyde exposure has been shown to lead to the formation of DNA damage comprising of DNA-protein crosslinks (DPCs), base adducts and interstrand or intrastrand crosslinks. DPCs have recently drawn more attention because of recent advances in detection and quantification of these adducts. DPCs are highly deleterious to genome stability and have been shown to block replication forks, leading to wide-spread mutagenesis. Cellular mechanisms to prevent DPC-induced damage include excision repair pathways, homologous recombination, and specialized proteases involved in cleaving the covalently bound proteins from DNA. These pathways were first discovered in formaldehyde-treated cells, however, since then, various other aldehydes have been shown to induce formation of DPCs in cells. Defects in DPC repair or aldehyde clearance mechanisms lead to various diseases including Ruijs-Aalfs syndrome and AMeD syndrome in humans. Here, we discuss recent developments in understanding how aldehydes form DPCs, how they are repaired, and the consequences of defects in these repair pathways.

## Introduction

Aldehydes are reactive carbonyl compounds that are abundantly present in the environment, ambient air, and human diets. Human cells also generate endogenous aldehydes from a variety of sources. Because of their polar carbon-oxygen double bond, they can react with all the major biomolecules of the cell, including DNA. Notably, multiple aldehydes can form direct DNA base adducts and DNA-protein crosslinks (DPC). The sources, types, adducts, and mutagenic effects of aldehydes have all been thoroughly reviewed (1). DPCs are a highly variable class of DNA lesions that can vary in size depending on the identity of the crosslinked protein. These bulky adducts pose a significant threat to DNA-based processes and have the ability to block transcription if the DPC is located on the template strand (2). DPCs can also block replication fork progression when they are located on the leading strand by inhibiting CMG helicase movement (3, 4). Finally, DPCs may inhibit new DNA synthesis by blocking DNA polymerases and have been shown to interfere with chromatin remodelling (5–7). Overall, various studies have shown that DPCs are an extremely heterogenous class of DNA damage that can have different impacts on the genome depending on the identity and location of the crosslinked protein (8, 9).

As evidence of the importance of DPC repair in maintaining genomic stability, mutations in *SPRTN*, encoding a specilized DPC protease, have been shown to be the cause of Rujis-Aalfs syndrome. This genetic disorder leads to genomic instability, early-onset hepatocellular carcinoma, and progeria (10, 11). This cancer predispoisiton phenotype in the absence of efficient DPC processing underscores how common and damaging these lesions are in the genome.

The goal of this review is to summarize which aldehydes generate DPCs and how these adducts are formed. We will also review how these DPCs are repaired, the consequences of improper repair, and avenues for future work in the field.

## Aldehydes as sources of DPCs- formaldehyde, acetaldehyde, acrolein, methylglyoxal, and malondialdehyde

### Formaldehyde

**Figure 1:**
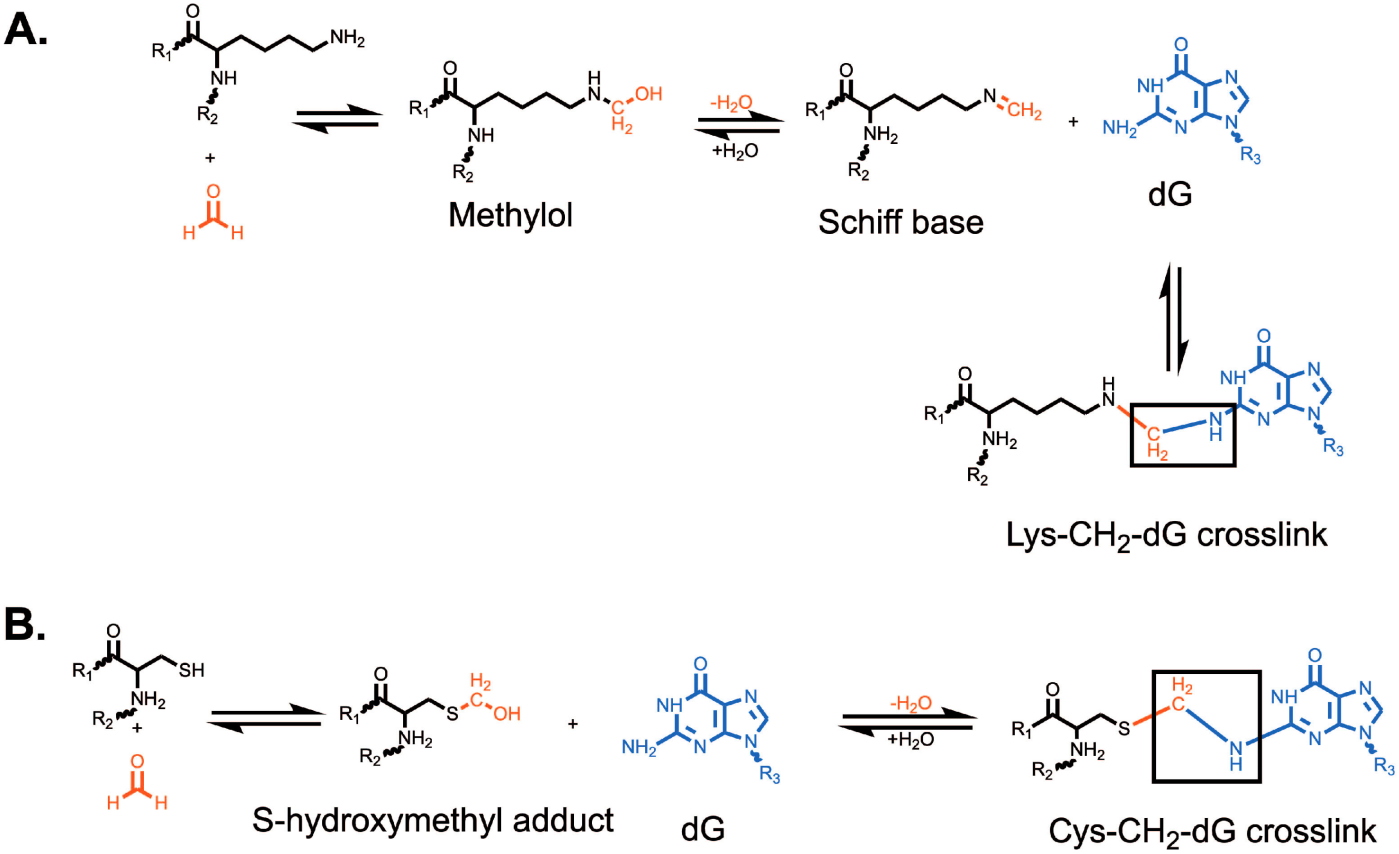
Formation of formaldehyde-induced **(A)** lysine-deoxyguanosine and **(B)** cysteine-deoxyguanosine crosslinks. Formation of a lysine-deoxyguanosine crosslink requires the formation of a methylol followed by dehydration to form a reactive Schiff base. The Lys-CH_2_-dG crosslink is the most common formaldehyde-induced crosslink. Formation of the cysteine-deoxyguanosine crosslink requires the formation of a S-hydroxymethyl adduct which can then directly react with deoxyguanosine. The Cys-CH_2_-dG crosslink is the most stable formaldehyde-induced crosslink. R_1_ and R_2_ represent the attachment of lysine **(A)** or cysteine **(B)** to a larger polypeptide chain. R_3_ represents the attachment of guanine to a deoxyribose group and DNA strand.

Formaldehyde is environmentally abundant from sources such as vehicle exhaust, factories, cigarettes, nail salons and is formed upon consumption of aspartame or methanol (12, 13). It is also produced endogenously in close proximity to DNA by processes such as histone demethylation (14, 15) and repair of DNA-methylation damage by AlkB-family dioxygenases (16, 17). Finally, it can be generated via one-carbon metabolism (18). Formaldehyde is highly reactive towards nucleotides and proteins, generating various DNA adducts which contribute to genomic instability (19, 20). Amongst the most studied adducts induced by formaldehyde are DPCs. Formaldehyde generally creates crosslinks by forming a covalent bond with a nucleophilic group, such as amines, amides, thiols, and hydroxyls, which generates a methylol adduct (21). This methylol can then be converted into a Schiff base via a dehydration reaction. These Schiff bases are unstable and either decompose or are stabilized when they react with a nucleophilic group in an adjacent molecule, forming a methylene bridge that links the two molecules (21). Formaldehyde has been shown to react with cysteine, histidine, lysine, tryptophan, and arginine (22). The most common formaldehyde-induced DPC occurs between deoxyguanosine and lysine, while the most stable DPC is between deoxyguanosine and cysteine (21) (**Figure 1**). It has been suggested that formaldehyde can only crosslink proteins that interact with DNA for greater than 5 seconds (23). For this reason, lysine and arginine-rich histones have been identified as the most common formaldehyde crosslinked proteins (24–26). Other commonly crosslinked proteins include topoisomerases and polymerases (26).

Formaldehyde-induced DPCs have been identified in cultured cells, animals, and humans exposed to formaldehyde. Formaldehyde is a known carcinogen and has been shown to be involved in the etiology of nasopharyngeal cancer in humans and squamous cell carcinomas in the nasal respiratory epithelium of rodents (27–29). Various aldehyde dehydrogenases (ALDH) and enzymes that process aldehyde-glutathione conjugates have been implicated in aldehyde clearance from cells (30). Mice carrying defects in alcohol dehydrogenase 5 (ADH5) and FANCD2 demonstrated formaldehyde-induced genotoxicity in hepatocytes, nephrons, and hematopoietic stem cells (31). Further, defects in ADH5 and ALDH2 led to elevated formaldehyde-induced DNA damage, mutagenesis, and carcinogenesis. Defective ADH5 and ALDH2 led to decreased clearance of endogenously formed formaldehyde leading to accumulation of this genotoxin (32). While these studies did not directly assay DPCs, it is likely that DPC formation was at least partly responsible for the observed genotoxicity. In an effort to determine the relative contribution of endogenous and exogenous formaldehyde in generating DPCs, rats were exposed to isotope labeled (^13^CD_2_]-formaldehyde) formaldehyde and various tissues were subjected to mass spectrometry (33). The researchers observed an increase in labeled exogenous DPCs in the nasal respiratory tissues but found no labeled DPCs in other tissues (33). Endogenous formaldehyde-induced DPCs were present in all the tested tissue types. This data suggests that endogenous formaldehyde, which is present in human blood at a concentration of ~100 µM, could be responsible for a vast majority of DPCs (33, 34). Similarly, DPCs were detected in the respiratory tract of rhesus monkeys exposed to formaldehyde (35). Finally, elevated DPC levels were detected in lymphocytes obtained from workers in hospital pathology departments who had chronic occupational exposure to formaldehyde (36). Such DPCs likely underlie formaldehyde-induced genotoxicity and carcinogenicity.

### Acetaldehyde

Humans are exposed to acetaldehyde from cigarette smoke and alcohol consumption. Acetaldehyde can react readily with deoxyguanosine to generate N^2^-ethylidene-deoxyguanosine (37, 38). While acetaldehyde can form DPCs, they have been shown to be unstable under physiological conditions. Treatment of histone-bound plasmids with acetaldehyde for one hour at 37˚C demonstrated an increase in DPCs; however, only 25% of crosslinks persisted past eight hours with an average half-life of 1.5-2 hours (39, 40). Nonetheless, defective DPC repair in fission yeast led to increased acetaldehyde-induced genotoxicity (41). Similarly, we recently demonstrated that deletion of DPC repair proteins in budding yeast leads to increased acetaldehyde-induced cytotoxicity and mutagenesis (42). These data indicate that despite the relatively low stability of acetaldehyde-induced DPCs *in vitro*, these moieties are potential sources of genomic instability *in vivo*.

### Acrolein

Acrolein, or 2-propenal, is abundantly present in cooked foods and the environment. Some common sources of acrolein include tobacco smoke, lipid metabolism, and fossil fuel combustion. It can also be produced endogenously from methionine and threonine synthesis (43). Acrolein reacts readily with nucleophiles such as deoxyguanosine to make mostly α- and γ-hydroxy-1,N^2^-propano-2’-deoxyguanosine (1, 43). These adducts have been shown to promote the formation of DNA interstrand crosslinks and have been used *in vitro* to generate acrolein-induced peptide adducts (44, 45). Finally, acrolein has been shown to react with the thiol group of cysteine, the imidazole group of histidine, and the amino groups of lysine and arginine to form Schiff bases and crosslinks (43, 46, 47).

### Methylglyoxal

Methylglyoxal, also known as pyruvaldehyde or 2-oxo-propanal, is an oxo-aldehyde that is generated endogenously from triose phosphate precursors during respiration (1, 48). Humans are also exposed to methylglyoxal from food and beverages, cigarette smoke, and vehicle exhaust (48). Methylglyoxal generates DNA adducts on deoxyguanosine and deoxyadenosine, and has an ability to react with arginine, lysine, and cysteine (48, 49). The protein and DNA adducts formed by methylglyoxal are referred to as advanced glycation end-products (AGEs), which have been implicated in many diseases such as cancer, diabetes, and various neurodegenerative diseases (1, 50, 51). Methylglyoxal is a bis-electrophile, meaning that it can form crosslinks between two independent nucleophiles through two electrophilic centers. Previously, it has been shown to form crosslinks *in vitro* between DNA and the *E. coli* DNA polymerase I (52). In this study, crosslinks were only observed between 2’-deoxyguanosine and *Nɑ-*acetyllysine or *N-*acetylcysteine, suggesting that methylglyoxal specifically makes deoxyguanosine-lysine or deoxyguanosine-cysteine DPCs (52).

### Malondialdehyde

Malondialdehyde is produced by polyunsaturated fatty acid peroxidation (53–55). Under physiological conditions, malondialdehyde is an enolate anion and is of relatively low reactivity (56). Despite this, it has been shown experimentally to produce DNA base adducts which are typically found on deoxyguanosine and deoxyadenosine (57, 58). Malondialdehyde has been shown to crosslink histones to DNA (59). Histone H1 was found to have an especially high affinity for malondialdehyde-induced crosslinking, and the authors concluded that this was likely due to the abundance of lysine residues within histone H1 (59). Importantly, these crosslinks formed readily at physiological temperature and pH, and were stable for up to 13 days. Malondialdehyde crosslinking was found to be limited to proteins that physically bind DNA, as a control BSA protein was unable to form crosslinks regardless of the concentration of malondialdehyde (59).

## Repair of aldehyde-induced DPCs

Various studies *in vitro* and in model systems have shown that DPCs block DNA polymerases (5, 60–62). Moreover, treatment of V79 Chinese hamster cells with formaldehyde led to elevated levels of sister chromatid exchange and micronuclei formation, likely due to abundant DPCs (63). These data indicate that DPCs formed by formaldehyde lead to genomic instability, which significantly contributes to formaldehyde-induced carcinogenesis. As such, mechanisms to remove DPCs are conserved from yeast to mammals. These mechanisms will be reviewed in the following section.

## Proteolysis-dependent DNA-protein crosslink repair

### Yeast Wss1 and Ddi1

**Figure 2:**
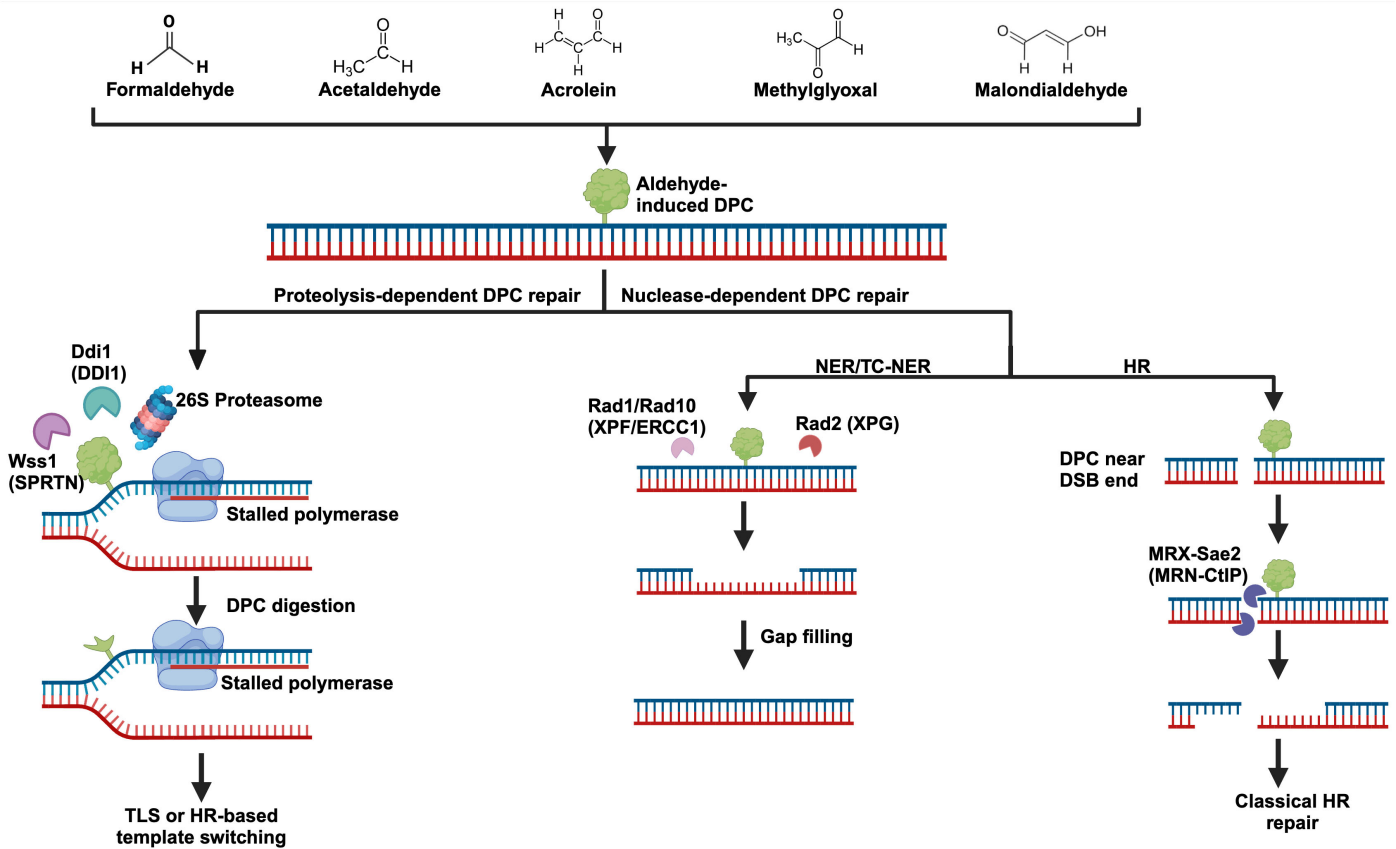
Aldehyde-induced DNA-protein crosslinks and their repair. Several biologically significant aldehydes can generate DPCs, and their structures can be seen at the top of the figure. DPC repair can be carried out by dedicated proteases like Wss1 (SPRTN), Ddi1, and the proteasome. DPCs may also be repaired by more classical pathways including nucleotide excision repair (NER) and homologous recombination (HR). Importantly, DPC repair most likely involves a combination of these pathways. For example, partial degradation of a DPC by the proteasome followed by NER to remove the remaining adduct. Yeast protein names are written with their human counterparts in parenthesis.

In 2014, Julian Stingele and Stefan Jentsch discovered and characterized the protease weak suppressor of Smt3 (Wss1) (64). This finding was significant as it was the first protease discovered to process DPCs regardless of the identity of the crosslinked protein in S-phase (**Figure 2**). Wss1 has a compact protease domain with an active site that contains few specificity-generating features (65). This general protease domain likely explains why Wss1 can process such a large variety of protein substrates. Wss1 becomes activated by DNA, and activation is most robust in the presence of single-stranded DNA (64). The requirement of DNA for Wss1 activation is an important regulator of function, as it limits Wss1’s protease activity to exclusively DPCs. Once activated, Wss1 targets SUMOylated DPCs via interactions with its SUMO-binding domain (8, 9). Finally, Wss1 function is dependent on its interaction with the chaperone-like enzyme Cdc48 which likely regulates Wss1 access to substrates (66). In the absence of this interaction, which is mediated by Ubx5, *in vivo* function of Wss1 is eliminated (66).

Enzymatic DPCs are common and can occur without any exogenous influence. For this reason, specific repair of commonly crosslinked DNA-interacting enzymes has evolved. Tyrosyl-DNA phosphodiesterase 1 and 2 (Tdp1 and Tdp2) have evolved to process topoisomerase crosslinks (67). Interestingly, Wss1 can process both enzymatic and non-enzymatic DPCs, and synthetic sickness has been observed in *Δtdp1Δwss1* yeast (64). This suggest that Wss1 may function in a secondary pathway to Tdp1 and Tdp2 in processing enzymatic DPCs. The *Candida albicans* Wss1 homolog, *Ca*Wss1, has been shown to protect against formaldehyde-induced DPCs (68). Finally, fission yeast with Wss1 or Wss2 deletions had an increased sensitivity to acetaldehyde and formaldehyde (41, 69). Together, these data suggest that Wss1’s role in the processing of DPCs is highly conserved.

Ddi1 is an aspartic protease that can interact with ubiquitylated proteins and the proteasome via its Ub-like domain (UBL) (70, 71). Humans possess a Ddi1 ortholog, and the sequence identity between human and yeast Ddi1 is 55% (72, 73). In humans, it is thought to promote proteasome-dependent replication fork restart following replication stress by degrading the replication termination factor 2 (72, 74). A role for the human DDI1 in DPC repair has not yet been established. In yeast however, Ddi1 has been shown to aid in the processing of camptothecin and formaldehyde-induced DPCs (**Figure 2**) (71, 75). While Ddi1 has a similar function to Wss1 in yeast, DPC processing by Wss1 seems to be the primary pathway. In the absence of the Ubx5-Cdc48-Wss1 axis, yeast become more dependent on Ddi1, and deletion of Wss1 results in an increase in Ddi1 protein levels (66, 71). Accordingly, *Δwss1Δddi1* yeast have been shown to be extremely sensitive to crosslinking agents (71). Deletion of either Wss1 or Ddi1 leads to increased acetaldehyde mutagenesis, and *Δwss1Δddi1* strains had a further increase in mutagenesis (42).

### SPRTN (DVC1) and other proteases in higher eukaryotes

Shortly after the discovery of the role of Wss1 in yeast, a similar DPC repair mechanism was identified *in vitro* using *Xenopus laevis* egg extracts (76). In this study, Duxin et al. found that replication coupled proteolysis could prevent persistent replication fork stalling at a DPC (76). In 2015, reciprocal BLAST searches were performed to find proteins with sequence similarity to Wss1 (9). The human protein SPRTN was found to be a match, and a phylogenetic analysis based on sequence similarity and domain organization found that they shared N-terminal protease domains and C-terminal tails containing motifs for binding Cdc48/P97/VCP (9). Further, both SPRTN and Wss1 can bind ubiquitin or SUMO, and SPRTN contains a PIP box for interactions with PCNA, supporting the idea that SPRTN associates at the replication fork (9). Despite the obvious similarities of these two proteins, they share very little sequence homology, and it is most likely that their similarity is due to convergent evolution. As such, SPRTN is generally considered to be a functional homolog of Wss1. In 2016, SPRTN was finally shown experimentally to be the mammalian protease responsible for processing DPCs in cells (77, 78).

To prevent promiscuous cleavage of DNA bound proteins, SPRTN function is tightly regulated. This regulation is dependent on ubiquitylation, the type of DNA substrate, and autocleavage (78). Firstly, mono-ubiquitinated SPRTN is excluded from chromatin (78). Upon induction of DPCs by formaldehyde treatment, SPRTN gets deubiquitinated and it can now return to chromatin (78). SPRTN activity is also strongly regulated by DNA-structure specificity. More specifically, it can process crosslinks at or around disruption in the double-stranded DNA helix (79). Examples of these disruptions include short ssDNA gaps, double-stranded breaks, mispaired DNA bubbles, or ssDNA-dsDNA junctions (79). Notably, SPRTN is unable to process crosslinks on strictly dsDNA or ssDNA substrates. This specificity is determined by SPRTN containing both a basic DNA-binding region (BR) and a zinc-binding domain (ZBD) (79). The BR domain preferentially binds dsDNA while the ZBD domain binds ssDNA, and proper SPRTN association and activation on DNA is dependent on both regions binding (79). Finally, SPRTN undergoes autocleavage as a negative regulator to its activity (78).

SPRTN function is essential to genome maintenance in higher eukaryotes. *Sprtn^-/-^* MEF cells have higher levels of γH2Ax, 53BP1, and RAD51 foci, as well as increased CHK2 activation (77, 78, 80). These results illustrate the importance of SPRTN in processing DPCs to prevent replication stress and subsequent double-stranded breaks (**Figure 2**). SPRTN expression is tightly regulated to S and G2 phases of the cell cycle where it can associate and travel with replication forks to prevent stalling at DPCs (81). Recently, it was shown that the helicase FANCJ is essential in promoting SPRTN function by binding to ssDNA downstream of the DPC and unfolding the protein adduct (82). This unfolding exposes the DNA beneath the crosslink, enabling SPRTN cleavage of the DPC (82). This cleavage frees most of the crosslink from the DNA but leaves a short peptide tail which must be bypassed by translesion synthesis. During G1, SPRTN gets degraded by APC/Cdh1 (81). It is thought that outside of S and G2, the proteasome may dominate in processing DPCs (83).

Mutations within *SPRTN* have been shown to be the cause of Ruijs-Aalfs syndrome, a genetic disorder that leads to genomic instability, early-onset hepatocellular carcinoma, and progeria (10, 11). Unsurprisingly, lymphoblastoid cells derived from patients with Ruijs-Aalfs syndrome had increased levels of γH2Ax staining compared to control cells following formaldehyde exposure (77). The characterization of Ruijs-Aalfs syndrome has underscored the importance of SPRTN in maintaining genomic stability and the effect of aldehyde-induced DPCs when they cannot be properly repaired.

Higher eukaryotes also contain the serine protease FAM111A which can repair TOP1 and PARP1-DNA crosslinks (84). Knockout of *FAM111A* resulted in only a slight sensitivity to formaldehyde, suggesting that either it only repairs a small portion of DPCs or that it works in a secondary pathway to SPRTN (84). In support of this idea, work from another group found that SPRTN, and not FAM111A, was responsible for processing PARP1-DNA crosslinks (85). Saha et al. concluded that these differing results were likely due to differential expression of FAM111A among different cell types where a lower SPRTN expression in one cell type may be compensated with higher FAM111A expression. Importantly, FAM111A expression is highest in late S phase (86).

Finally, the acidic repeat containing protein (ACRC/GCNA) was found in higher eukaryotes and was determined to contain a SprT domain like SPRTN and Wss1, and is similar to SPRTN based on phylogenetic analysis (83, 87, 88). ACRC/GCNA has been shown to interact with polySUMO chains and localizes to formaldehyde-induced damage foci (89). ACRC/GCNA is required for germ cell genomic stability in *Drosophila, C. elegans*, and zebrafish (90). ACRC/GCNA seems to be especially well suited to resolving topoisomerase II DPCs (90, 91). Despite these discoveries, the roles of ACRC/GCNA in DPC repair remains largely unexplored.

### Proteasomal degradation of aldehyde-induced DPCs

The 26S eukaryotic proteasome is an extremely large ATP-dependent protease that is recruited to several cellular locations to degrade proteins (92). For efficient degradation, proteins must pass into the hollow 20S proteasomal core (7). Essential to the regulation and function of the proteasome is its ability to recognize polyubiquitylated protein substrates using specialized ubiquitin binding sites (92). The proteasome is intimately related to the DPC-repair response (**Figure 2**). Inhibition of the 26S proteasome in A549 cells resulted in a significant reduction in formaldehyde-induced DPC repair (93), and sensitized cells to formaldehyde treatment (94). In *Xenopus laevis* egg extract, proteasome components were found to accumulate on replicating plasmids containing DPCs, and degradation of DPCs was dependent on polyubiquitylation by the E3 ligase TRAIP (95). Proteasomal and SPRTN mediated processing of DPCs are non-redundant pathways. When SPRTN was depleted in *Xenopus* extract, translesion synthesis across the DPC was impaired even when the proteasome was unchanged, suggesting that SPRTN was the preferred pathway to process the crosslink (95). Given that DPCs can not readily enter the proteasomal 20S core, there are a few models for how it may contribute to DPC proteolysis. Firstly, it is possible that the proteasome partially degrades a DPC into a smaller peptide crosslink which may be removed by nucleotide excision repair (7). It is also likely that proteasomal degradation depends on specialized proteases such as SPRTN or TDP1 to cleave the bulk of a DPC off of DNA, with the now freed protein being a more manageable substrate for the proteasome (7). Because aldehydes can generate a heterogenous class of DPCs, it is most likely that coordination between different repair pathways are required to efficiently resolve aldehyde-induced DPCs (**Figure 2**).

## Nuclease-dependent DNA-protein crosslink repair

### Global nucleotide excision repair

Nucleotide excision repair (NER) can recognize helix distorting lesions and involves the removal of the lesion and surrounding nucleotides, leaving a single-stranded DNA gap (**Figure 2**). Early studies in bacteria showed that NER was involved in processing formaldehyde-induced DNA damage as *uvrA* mutants showed an increased sensitivity (96). Studies in yeast have shown that NER defective strains (*Δrad1* and *Δrad4)* were highly sensitive to acute formaldehyde treatment (60 mM for 15 minutes), but were not especially sensitive to chronic formaldehyde treatment (1.5 mM for 48 hours) (97). Similarly, deletion of the NER pathway in fission yeast led to increased sensitivity of cultures to formaldehyde and acetaldehyde (41, 69). These data were echoed by our recent study demonstrating a role for NER in the repair of acetaldehyde-induced lesions (42). These results suggest that NER is an important ‘first line of defense’ to aldehyde-induced DPCs, but if given adequate time, other repair pathways can compensate for loss of NER. In human cells, NER was able to repair partially digested DPCs but was unable to repair certain full sized DPCs (98). In fact, mammalian NER is limited to relatively small 8-10 kDa (~70-90 amino acids) crosslinks (99). For this reason, it is likely that NER works cooperatively with the DPC proteases described earlier in this review, where the DPCs are cleaved and the remaining peptide tail is then removed by NER in a subsequent step.

### Transcription-coupled repair of aldehyde-induced DPCs

Recently, it was shown that transcription-coupled repair is involved in processing formaldehyde-induced DPCs. Cells were treated with 600 µM formaldehyde for one-hour followed by zero- and four-hour recovery times. After four hours, there was a marked reduction in DPC-seq derived sequencing reads within gene bodies (100). This result suggests that DPCs within transcribed regions are being efficiently repaired. To further support the hypothesis that transcription promotes DPC repair, the RNA polymerase II transcribed genes *PKM, TK1, AFMID*, and *TCF7L2* were analyzed in cells treated with an RNA polymerase II inhibitor. Upon inhibition of RNA polymerase II, there was a reduction in DPC processing within the targeted gene bodies, suggesting that crosslink repair was dependent on active transcription (100). Immunoprecipitation of elongating RNA polymerase II in the presence of formaldehyde followed by mass spectrometry showed that RNA polymerase II was associated with the transcription-coupled nucleotide excision repair factors CSA, CSB, UVSSA, and TFIIH (100). In the absence of CSB, cells were unable to recover RNA synthesis, suggesting that transcription-coupled repair is required for rescuing DPC-stalled transcription machinery (100). Overall, this work establishes a novel role for transcription-coupled nucleotide excision repair in the processing of formaldehyde-induced DPCs. Whether other aldehyde-induced DPCs can be repaired via a similar transcription-coupled mechanism remains to be seen. In the future, it would be interesting to test if proteasomal or SPRTN function is required for the DPC-processing role of transcription-coupled nucleotide excision repair, and whether this pathway is under the same DPC size restrictions as global nucleotide excision repair.

### Homologous recombination in DPC repair

Early studies in bacteria showed that *recA* mutants were sensitive to formaldehyde treatment, establishing a role for homologous recombination (HR) in the repair of aldehyde-induced damage (96). Interestingly, HR in yeast was shown to aid DPC processing following chronic, low dose exposure to formaldehyde while not contributing significantly to the repair of acute, high dose treatment (97). HR was also shown to be required for tolerance to acetaldehyde and formaldehyde in fission yeast (41, 69). HR has been implicated in the processing of DPCs in higher eukaryotes as well. A study in chicken DT40 cells that were defective in the BRCA/FANC pathway and HR were hypersensitive to low doses of formaldehyde and acetaldehyde, but were not sensitive to acrolein, crotonaldehyde, glyoxal, or methylglyoxal (101). There are a few ways in which HR may contribute to DPC repair. Firstly, HR may assist in the processing of DPCs in close proximity to double-stranded breaks with the help of the MRE11-RAD50-NBS1 (Xrs2 in yeast) complex (**Figure 2**) (102). The MRN/MRX complex has been shown to remove Spo11-generated crosslinks from the ends of a double-stranded break in yeast and in mouse testis extract, confirming that DPCs can be efficiently processed from double-stranded break ends (103). Finally, *Xenopus* egg extracts have been used to show that MRN, CtIP, and BRCA1 work together in the removal of TOP2-DPCs at double-strand break ends (104), and very similar results have been observed in mammalian cells (105, 106). HR may also assist in DPC tolerance via the template switching pathway. When forks stall at a DPC, the sliding clamp PCNA can be polyubiquitinated which leads to template switching initiation (107). In this model, the stalled polymerase uses the nascent sister strand as a template, allowing for extension beyond the adduct. The resulting structure can then be resolved via a classic HR dissolution reaction (107, 108). It is important to note that template switching is a DNA damage tolerance pathway, and following bypass the DPC will persist and must be repaired later. Given that aldehydes generate DPCs which can lead to double-stranded breaks if they persistently block replication forks, it is likely that homologous recombination plays a significant role in processing DPCs both at break ends and via template switching to restore chromosomal integrity.

In summary, efficient nucleotide excision repair depends on the recruitment of various bulky protein complexes. As such, DPCs can only be repaired by NER if they do not impede the recruitment of the NER factors. It is possible that NER works in concert with DPC proteases or the proteasome, wherein the proteases partially process the DPC, enabling NER to excise the remaining peptide bound DNA. Importantly, DPC processing by NER can likely occur throughout the cell cycle. HR does not face the same size constraints as NER, but generally can only resolve crosslinks near double-stranded breaks using the nuclease activity of the MRN complex. Repair of DPCs by HR requires the presence of a double-strand break and homologous DNA, likely limiting this repair to S/G2 phases of the cell cycle. Given that aldehydes can crosslink a variety of proteins to DNA, it is likely that an elaborate combination of protease- and nuclease-based repair pathways are required for efficient processing of aldehyde-induced DPCs. A graphical summary of protease- and nuclease-dependent DPC repair pathways can be seen in **Figure 2**.

## DNA-protein crosslink-induced genotoxicity in cancers

ALDH2 is a key enzyme that removes acetaldehyde and various other endogenous aldehydes from the cell. Defects in ALDH2 have been linked to increased alcohol-associated cancers (109). Interestingly, low ALDH2 levels are also predictive for poor survival in lung and liver cancer patients, likely due to elevated cellular aldehydes. Inhibition of ALDH2 has been shown to cause an increase in DPCs in cancer cell lines (110). As such, DPC-induced genome instability from endogenous aldehydes is common in cancers with defective aldehyde clearance pathways.

Germline mutations in SPRTN have been seen in patients with Ruijis-Aalfs syndrome which is characterized by increased risk of hepatocellular carcinoma (10, 111). SPRTN hypomorphic mice were also shown to have elevated levels of DNA damage, DPCs, and spontaneous development of liver tumors (112). These studies demonstrate that the liver is the primary site of carcinogenesis in patients with SPRTN deficiencies. However, why these patients develop liver cancers has remained unclear. We hypothesize that lipid peroxidation by cytochrome P450 enzymes in the liver lead to elevated cellular aldehydes and DPCs.

SPRTN was shown to be involved in the unfolded protein response (UPR) and interacts with GRP78 in HepG2 liver cancer cells (111). SPRTN depleted cells exhibit increased sensitivity to ER stress and the UPR. Increased ER stress was shown to lead to DNA damage that was epistatic to DNA damage induced by SPRTN deficiency (111). It could thus be possible that increased ER stress leads to DNA damage that is repaired via SPRTN-induced proteolysis. ER stress and the UPR also results in fatty acid accumulation in cells (113). Cytochrome P450 enzymes have been shown to directly lead to oxidation of lipids forming lipid hydroperoxides (114). Moreover, activity of the cytochrome P450 enzymes leads to elevated free radical generation that can further lead to lipid peroxidation (115). Lipid peroxidation in liver cells is expected to generate reactive aldehydes like malondialdehyde, 3-hydroxynonenal, acrolein, and acetaldehyde (54, 55, 116). It is likely that the reactive aldehydes produced upon cytochrome P450 mediated lipid peroxidation in hepatocytes induce DPCs which are targets for SPRTN processing. In the absence of SPRTN, these persistent DPCs lead to DNA damage and mutagenesis, likely contributing to the development of hepatocellular carcinoma in patients with Ruijis-Aalfs syndrome.

Alternately, livers from *SPRTN* hypomorphic mice demonstrate an accumulation of topoisomerase I cleavage complexes (112). Such adducts also lead to acute DNA damage and genome instability culminating in carcinogenesis. Interestingly, lipid peroxidation products including 4-hydroxynonenal have been shown to modify TOP1 active site residue C630 leading to TOP1 entrapment on DNA (117). In the absence of SPRTN, TOP1-DPCs would accumulate leading to genome instability.

These studies demonstrate the importance of SPRTN in mitigating DNA damage from aldehyde-induced DPCs in liver cancers.

## Conclusions

Aldehydes are present ubiquitously in the environment, and several are generated endogenously by cells. These aldehydes have been shown to generate a variety of DNA damage including base adducts, interstrand and intrastrand DNA crosslinks, and DPCs. DPCs can have a variety of deleterious effects on genome integrity; inhibiting DNA synthesis, transcription, and chromatin remodeling. As such mechanisms for repair of DPCs are evolutionarily conserved. These include proteolysis-dependent repair with the specialized proteases Wss1/SPRTN and Ddi1, or more general proteolysis using the proteasome. Cells can also employ nuclease-dependent repair of DPCs which include global and transcription-coupled nucleotide excision repair, as well as homologous recombination. Defects in any of these repair pathways greatly sensitize cells to aldehyde-induced DPCs, and evidence of this can be seen in Rujis-Aalfs syndrome, a cancer predisposition disorder that results from hypomorphic mutations in *SPRTN.*

Research on reactive aldehydes has been challenged by several technical limitations. Firstly, many of these aldehydes are highly volatile so maintaining consistent experimental conditions can be challenging. DPC research has long been limited by the ability to detect DPCs. Relatively new detection methods such as rapid approach to DNA adduct discovery (RADAR) and purification of x-linked proteins (PxP) promise to increase reliability and sensitivity of DPC detection (26, 118, 119). Finally, these reactive aldehydes can generate a variety of adducts, making it difficult to determine the impact of DPCs specifically. While the field of DPC repair has seen significant advances in recent years, several questions remain. Much of the work done on aldehyde-induced DPCs has used formaldehyde. It is still unknown if the damage generated by other aldehydes are processed by SPRTN in similar ways. While many studies have looked at double-stranded breaks following the loss of DPC repair, the mutagenic consequences of losing DPC repair are relatively unknown. Finally, it is not well understood why reactive aldehydes and SPRTN function are drivers for liver cancer specifically. It remains to be seen if aldehyde levels are higher in SPRTN deficient cells or if SPRTN deficiency in conjunction with non-alcoholic fatty liver disease cause increased DPC formation and hepatocellular carcinoma development.
